# chi-miR-487b-3p Inhibits Goat Myoblast Proliferation and Differentiation by Targeting IRS1 through the IRS1/PI3K/Akt Signaling Pathway

**DOI:** 10.3390/ijms23010115

**Published:** 2021-12-23

**Authors:** Ming Lyu, Xu Wang, Xiangyu Meng, Hongrun Qian, Qian Li, Baoxia Ma, Zhiying Zhang, Kun Xu

**Affiliations:** Key Laboratory of Animal Genetics, Breeding and Reproduction of Shaanxi Province, College of Animal Science and Technology, Northwest A&F University, Yangling, Xianyang 712100, China; lyuming@nwafu.edu.cn (M.L.); 15091371182@163.com (X.W.); mxiangyu2021@163.com (X.M.); 18821714328@163.com (H.Q.); lqsymltk@163.com (Q.L.); 18792898523@163.com (B.M.)

**Keywords:** chi-miR-487b-3p, *IRS1*, PI3K/Akt, goat myoblast, proliferation, differentiation

## Abstract

MicroRNAs (miRNAs) are endogenously expressed small noncoding RNAs and play critical roles in the regulation of post-transcriptional gene expression. Our previous study uncovered that chi-miR-487b-3p is widespread in different goat tissues, which is significantly higher in muscle, especially in lamb. Here, we demonstrate the role of chi-miR-487b-3p as a myogenic miRNA that regulates skeletal muscle development. chi-miR-487b-3p overexpression was demonstrated to significantly inhibit goat myoblast proliferation and differentiation, whereas chi-miR-487b-3p inhibition resulted in the opposite effects. Next, chi-miR-487b-3p was predicted to target the 3′UTR of insulin receptor substrate 1 (*IRS1*) gene by Target-Scan and miRDB. The results of dual-luciferase assay, RT-qPCR, and western blot all confirmed that *IRS1* might be a direct target of chi-miR-487b-3p as its expression was negatively regulated by chi-miR-487b-3p. siRNA silencing of *IRS1* further demonstrated significant inhibition on goat myoblast proliferation and differentiation, confirming the effect of *IRS1* downregulation by chi-miR-487b-3p in myogenesis. In addition, chi-miR-487b-3p knockout goat myoblast clones were generated using CRISPR/Cas9 technology, and we further illustrated that chi-miR-487b-3p regulates goat myoblast growth through the PI3K/Akt signaling pathway by targeting *IRS1*. Collectively, our work demonstrated that chi-miR-487b-3p is a potent inhibitor of skeletal myogenesis and provided new insights into the mechanisms of miRNA on the regulation of goat growth.

## 1. Introduction

Skeletal muscle is the most abundant tissue in mammals and plays an important role in body metabolism [1]. During the formation of skeletal muscle, mononucleated myoblasts expand, migrate, and differentiate into myoblasts, which is a complex process regulated by a group of myogenic regulatory factors (MRF). All these factors including myogenic factor 5 (*Myf5*), myogenic determination 1 (*MyoD1*), myogenin (*MyoG*), and myosin heavy chain (*MyHC*) are the key regulators of skeletal muscle development [2,3]. Therefore, further understanding of the skeletal muscle development process and the related molecular mechanisms will contribute to young lamb growth improvement and facilitate mutton goat breeding.

MicroRNAs are a class of evolutionarily conserved small RNAs that inhibit target gene expression at post-transcriptional level either by degrading or arresting the translation of specific messenger RNAs (mRNAs) [4,5]. The short single-stranded miRNA binds to the 3′ untranslated region (3′UTR) of the specific mRNA based on sequence homology, resulting in the degradation of target mRNA or repression of corresponding protein translation [6,7]. Up to now, a series of miRNAs have been verified to be involved in the regulation of myoblast proliferation and differentiation. Among these, miR-1, miR-133, miR-206, and miR-486 are key muscle-specific regulators involved in myogenesis [8,9,10]. In addition, skeletal muscle is also enriched with many ubiquitously expressed miRNAs including miR-24, miR-29, miR-125b, miR-181, and miR-214, which are also necessary for the regulation of muscle development [11,12,13,14,15]. Both muscle-specific and ubiquitously expressed miRNAs have been demonstrated as major regulators of fundamental biological processes [16,17]. Further study of functional miRNAs in goat muscle development remains necessary to improve our understanding of how the miRNA network regulates goat myogenesis.

Previous studies have reported that cluster 14q32.31 member miR-487b-3p is ubiquitously expressed and plays an important role in many biological processes such as cell growth, proliferation, and differentiation [18,19,20]. miR-487b-3p binds directly to *IRS1* 3΄UTR and inhibits reporter gene expression in primary rat and human arterial adventitial fibroblasts [18,20]. *IRS1* is one of the dominant acting regulators of cell proliferation and survival, which regulate signal transduction from the IGF-1 receptors to intracellular pathways [18,20,21]. Recently, several studies have shown that *IRS1* regulates myoblast growth and glucose metabolism via the phosphatidylinositol 3-kinase (PI3K)/Akt pathway [13,22]. However, the function of miR-487b-3p on goat myogenesis remains unknown.

In this study, we systematically investigated how miR-487b-3p regulates goat myoblast cell proliferation and differentiation. A series of experimental approaches including quantitative real-time polymerase chain reaction (qRT-PCR), western blot, cell counting kit-8 assay (CCK-8), 5-ethynyl-2′-deoxyuridine (EdU), cell cycle analysis, and dual-luciferase reporter assay were used to find the potential role of miR-487b-3p in goat myoblast proliferation and differentiation. Additionally, miR-487b-3p knockout (KO) primary goat myoblast clones were generated using the clustered regularly interspaced short palindromic repeats/CRISPR-associated protein 9 (CRISPR/Cas9) technology and our DsRed-Puror-eGFP (RPG) surrogate reporter-based screening [23,24,25]. Hence, we provided compelling evidence demonstrating miR-487b-3p as a potent inhibitor of myogenesis by downregulating *IRS1* expression while suppressing the PI3K/Akt signaling pathway. This finding provides new insights into miR-487b-3p function in primary goat myoblasts, which will expand our understanding of skeletal muscle growth regulation.

## 2. Results

### 2.1. miR-487b-3p Acts as a Candidate Regulator in Goat Skeletal Myogenesis

**Figure 1:**
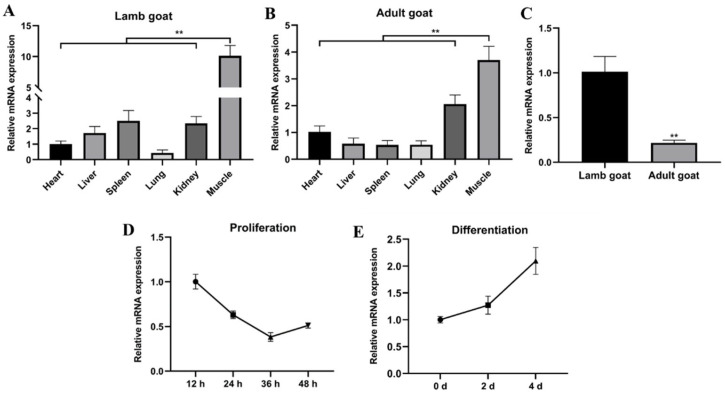
miR-487b-3p is a candidate regulator in goat myogenesis. (**A**) miR-487b-3p expression pattern in different lamb tissues. (**B**) miR-487b-3p expression pattern in different adult goat tissues. (**C**) Comparison of miR-487b-3p expression between lamb and adult goat muscle tissue. (**D**) miR-487b-3p expression pattern during goat myoblast proliferation. (**E**) miR-487b-3p expression pattern during goat myoblast differentiation. Data are shown by mean ± SEM of three independent experiments. ** *p* < 0.01.

To address the function of goat miR-487b-3p, miR-487b-3p expression pattern in different tissues was detected by qRT-PCR analysis. The data showed that miR-487b-3p was enriched in skeletal muscle compared with the heart, liver, spleen, lung, and kidney at both the lamb-stage and adult-stage (*p* < 0.01; Figure 1A,B). Furthermore, we noticed significantly higher miR-487b-3p expression in lamb goats than adult goats (*p* < 0.01; Figure 1C). We further found that miR-487b-3p expression decreased dramatically during proliferation (Figure 1D). Interestingly, miR-487b-3p expression reached the highest level after myogenic differentiation for four days (Figure 1E). These results suggest that miR-487b-3p might play a critical role in goat myogenesis.

### 2.2. miR-487b-3p Inhibits Goat Myoblast Proliferation

**Figure 2:**
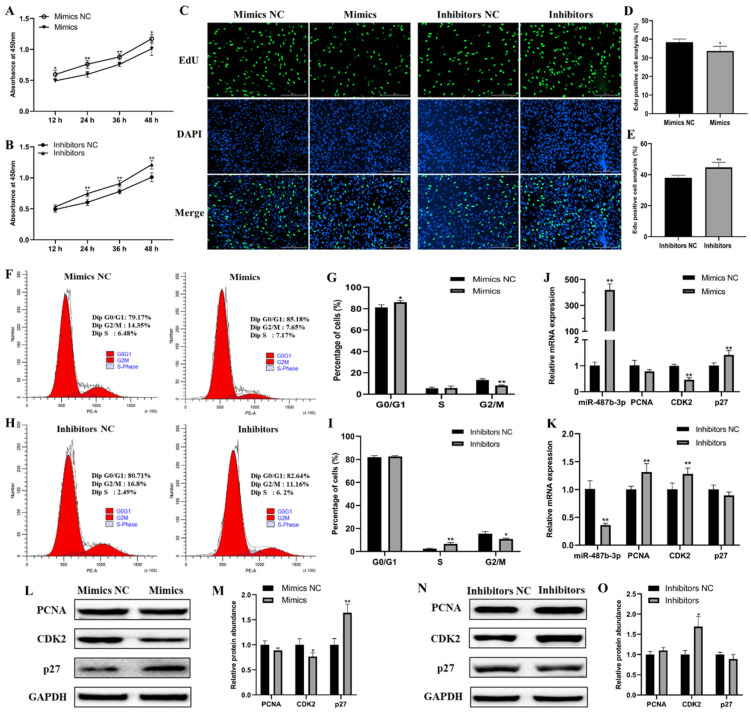
miR-487b-3p inhibits goat myoblast proliferation. (**A**,**B**) Goat myoblast proliferation detected by CCK-8 analysis after transfection with miR-487b-3p mimic, inhibitor, or negative control (NC). (**C**–**E**) Goat myoblast proliferation index detected by the EdU assay. Cells during DNA replication were stained by EdU (green) and cell nuclei were stained with DAPI (blue). The percentage of EdU positive cells/DAPI positive cells (total cells) was quantified. (**F**,**G**) Goat myoblast cell cycle analysis 48 h after miR-487b-3p overexpression. (**H**,**I**) Goat myoblast cell cycle analysis 48 h after miR-487b-3p inhibition. (**J**,**K**) qRT-PCR quantified expression of proliferation marker genes (*PCNA*, *CDK2* and *p27*) after the overexpression or inhibition of miR-487b-3p. (**L**,**M**) Western blot detected expression of proliferation marker genes following transfection with miR-487b-3p mimics or the negative control (Mimics NC). (**N**,**O**) Western blot detected expression of proliferation marker genes following transfection with miR-487b-3p inhibitors or the negative control (Inhibitors NC). Data are shown by mean ± SEM of three independent experiments. * *p* < 0.05; ** *p* < 0.01.

To explore the underlying effect of miR-487b-3p on goat myoblast proliferation, gain- and loss-of-function assays were conducted. The CCK-8 assay showed that miR-487b-3p overexpression inhibits cell proliferation in myoblasts (Figure 2A). In contrast, cell proliferation in the miR-487b-3p knockdown group was notably promoted (Figure 2B). Next, the EdU incorporation assay demonstrated that miR-487b-3p overexpression significantly inhibited myoblast proliferation (*p* < 0.05; Figure 2C,D), whereas the anti-miR-487b-3p group significantly promoted myoblast proliferation (*p* < 0.01; Figure 2C,E). The results of flow cytometric analysis showed increased population of G0/G1 phase cells and decreased G2/M phase cells in miR-487b-3p mimic transfected myoblasts (Figure 2F,G). miR-487b-3p inhibition elevated the percentage of S phase cells and reduced G2/M phase cells (Figure 2H,I). Further analyses revealed that miR-487b-3p overexpression or knockdown could regulate the expression of cell cycle-related genes (*PCNA*, *CDK2* and *p27*), both in the mRNA (Figure 2J,K) and protein (Figure 2L–O) levels. Taken together, all these results indicate that miR-487b-3p inhibits myoblast proliferation.

### 2.3. miR-487b-3p Inhibits Goat Myoblast Differentiation

**Figure 3:**
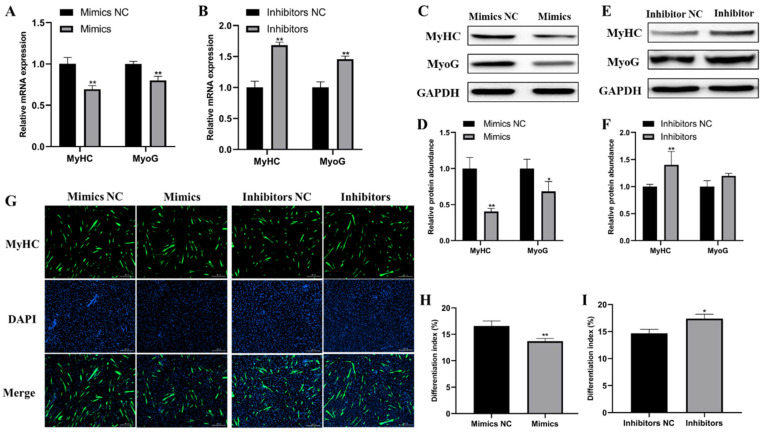
miR-487b-3p inhibits goat myoblast differentiation. (**A**,**B**) Myosin heavy chain (*MyHC*) and myogenin (*MyoG*) mRNA expression detected by qRT-PCR in differentiated cells transfected with miR-487b-3p mimics, miR-487b-3p inhibitors, or NC. (**C**,**D**) Western blot detected protein expression of MyHC and MyoG after miR-487b-3p overexpression. (**E**,**F**) Western blot detected protein expression of MyHC and MyoG after miR-487b-3p knockdown. (**G**–**I**) Differentiation index counted by MyHC-positive cells to total nuclei. Data are shown by mean ± SEM of three independent experiments. * *p* < 0.05; ** *p* < 0.01.

To confirm miR-487b-3p function on goat myoblast differentiation, we first measured the mRNA expression of myogenic genes including *MyoG* and *MyHC*. qRT-PCR results showed that miR-487b-3p overexpression significantly reduced the expression of *MyoG* and *MyHC* mRNAs, whereas their mRNA expression were significantly increased in miR-487b-3p knockdown myoblasts (*p* < 0.01; Figure 3A,B), which was consistent with the results of the protein expression detected by western blot (Figure 3C–F). Furthermore, the immunofluorescent assay demonstrated that goat myoblasts with miR-487b-3p overexpression had fewer myotubes and tended to have a lower differentiation rate (Figure 3G,H), while the opposite results were detected when miR-487b-3p was inhibited (Figure 3G,I). Overall, these data suggest that miR-487b-3p inhibits goat myoblast differentiation.

### 2.4. IRS1 Is a Direct Target of miR-487b-3p

**Figure 4:**
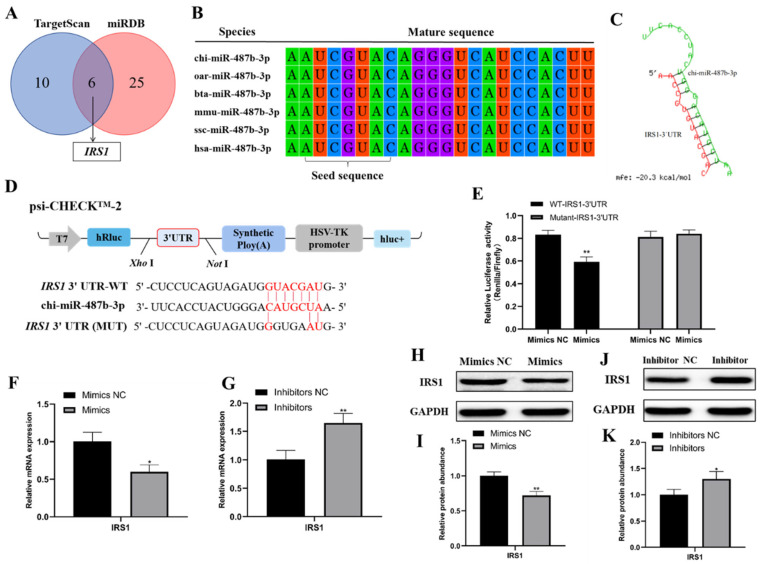
miR-487b-3p directly targets the *IRS1* gene. (**A**) miR-487b-3p target genes were explored using the online software TargetScan and miRDB. (**B**) Comparison of the miR-487b-3p sequences from *Ovis aries*, *Bos taurus*, *Mus musculus*, *Sus scrofa*, and *Homo sapiens*. (**C**) The RNA duplex structure of miR-487b-3p and *IRS1* 3′-UTR target site (Red: Target sequence; Green: chi-miR-487b-3p). (**D**) The illustration of the dual-luciferase reporter construct with goat *IRS1* 3΄UTR and the predicted wild type or mutant miR-487b-3p binding sites. (**E**) The results of the dual-luciferase reporter assay. Cells were co-transfected with the dual-luciferase reporter containing the wild type (WT) or mutant IRS1 3΄UTR, either with the negative control or miR-487b-3p mimics. The activity of renilla luciferase was normalized to firefly luciferase. (**F**,**G**) *IRS1* mRNA expression detected by qRT-PCR in goat myoblasts transfected with miR-487b-3p mimics, miR-487b-3p inhibitors, or NC. (**H**,**I**) IRS1 protein expression in goat myoblasts transfected with miR-487b-3p mimics or mimics NC. (**J**,**K**) IRS1 protein expression in goat myoblasts transfected with miR-487b-3p inhibitors or inhibitors NC. Data are shown by mean ± SEM of three independent experiments. * *p* < 0.05; ** *p* < 0.01.

To explore the molecular mechanism of miR-487b-3p on goat myoblast development, its potential target genes were predicted using the online software TargetScan and miRbase, and the *IRS1* gene was chosen as the preferred candidate for further study (Figure 4A). Subsequent analysis found that miR-487b-3p is located on chromosome 21 and is composed of 22 nucleotides. The mature miR-487b-3p sequence is highly conserved among several species including *Ovis aries*, *Bos taurus*, *Mus musculus*, *Sus scrofa*, and *Homo sapiens*, according to the miRBase and NCBI database (Figure 4B). Then, we used the RNA hybrid to analyze the duplex and the minimum free energy (mFE between miR-487b-3p and *IRS1* 3′-UTR). mFE of the RNA duplex was about −20.3 kCal/mole, indicating it has a high stability (Figure 4C). To confirm the miRNA–target relationship between miR-487b-3p and *IRS1*, the dual-luciferase reporter system was constructed. The 3΄UTR fragments from the *IRS1* gene containing the predicted wild type or mutant miR-487b-3p binding sites as shown in Figure 4D were cloned into the psi-CHECK-2 vector. The relative luciferase activity of the wild type 3′UTR group (WT-*IRS1*-3′UTR) was significantly inhibited as they responded to miR-487b-3p mimics (*p* < 0.01; Figure 4E), while no changes were observed in cells co-transfected with the mutated reporter (Mutant-*IRS1*-3΄UTR) (Figure 4E). In addition, *IRS1* mRNA expression was significantly downregulated by miR-487b-3p overexpression (*p* < 0.05; Figure 4F) and upregulated after miR-487b-3p knockdown (*p* < 0.01; Figure 4G). Next, similar alteration of IRS1 protein expression was detected (Figure 4H-K). These results suggest the direct target relationship between *IRS1* and miR-487b-3p.

### 2.5. IRS1 Knockdown Suppresses Goat Myoblast Proliferation and Differentiation

**Figure 5:**
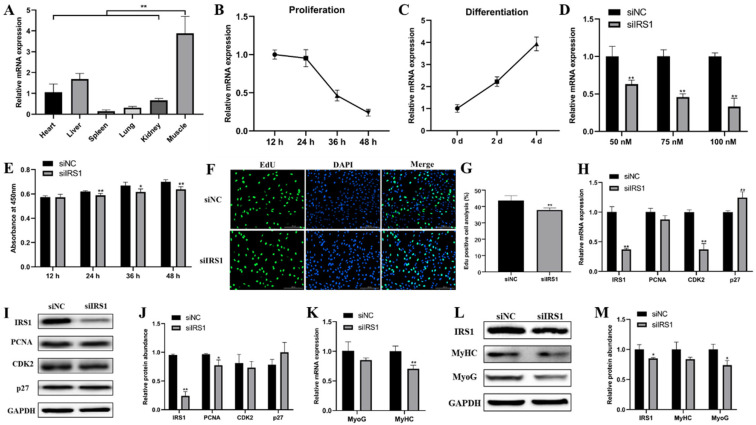
IRS1 knockdown suppresses goat myoblast proliferation and differentiation. (**A**) *IRS1* expression pattern in different lamb tissues. (**B**,**C**) *IRS1* expression profiles during goat myoblast proliferation and differentiation. (**D**) qRT-PCR quantified *IRS1* mRNA expression in goat myoblasts transfected with different siRNA concentrations. (**E**) Cell counting detected by CCK-8. (**F**,**G**) The cell proliferation index detected by the EdU assay. (**H**–**J**) qRT-PCR and western blot analyses of the expression of proliferation marker genes (*PCNA*, *CDK2*, and *p27*) in goat myoblasts transfected with siIRS1 or siNC. (**K**–**M**) qRT-PCR and western blot analyses of the expression of myogenic marker genes (*MyHC* and *MyoG*) in goat myoblasts transfected with siIRS1 or siNC. Data are shown by mean ± SEM of three independent experiments. * *p* < 0.05; ** *p* < 0.01.

We further examined the role of *IRS1* during goat myoblast proliferation and differentiation by RNA interference. First, we found that the *IRS1* gene is highly expressed in lamb-stage muscle tissues (Figure 5A) and has the opposite expression profiles during myoblast proliferating and differentiation (Figure 5B,C). The optimal siRNA transfection concentration in goat myoblasts was 100 nM, and siIRS1 demonstrated the best knock-down efficiency (*p* < 0.01; Figure 5D). Cell proliferation was detected by CCK-8 and EdU staining analysis. We found that *IRS1* knockdown significantly inhibited goat myoblast proliferation (Figure 5E–G). Furthermore, *IRS1* knockdown resulted in significant changes in the expression of cell cycle-related genes (*PCNA*, *CDK2*, and *p27*) at both mRNA and protein levels (Figure 5H–J). In addition, we also detected the effect of *IRS1* knockdown on myoblast differentiation. Compared with the siNC control, siIRS1 decreased the expression of differentiation genes (*MyoG* and *MyHC*) at both the mRNA and protein levels (Figure 5K–M). These results indicate that *IRS1* silencing represses goat myoblast proliferation and differentiation.

### 2.6. miR-487b-3p Affects the IRS1/PI3K/Akt Signaling Pathway

**Figure 6:**
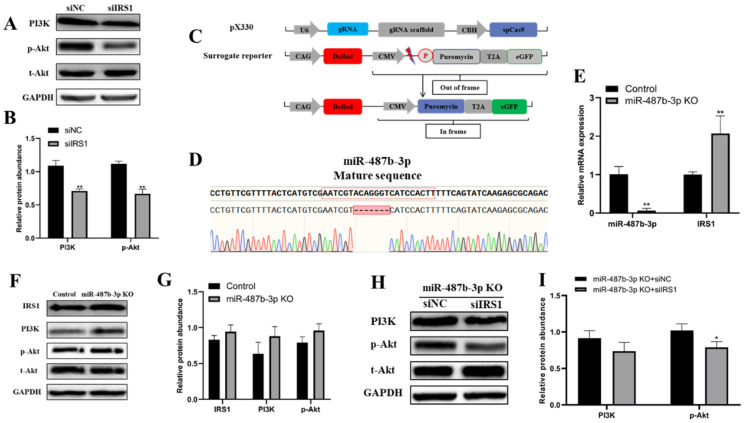
miR-487b-3p affects the IRS1/PI3K/Akt signaling pathway. (**A**,**B**) The protein expression of PI3K, phosphor-Akt (p-Akt), and total Akt (t-Akt) in goat myoblasts transfected with *IRS1* siRNA and control siRNA. (**C**) Schematic diagram of the DsRed-Puro^r^-eGFP (RPG) surrogate reporter screening system including a sgRNA/Cas9 expression vector and a RPG surrogate reporter vector. (**D**) The sequencing results of the miR-487b-3p KO myoblast clone. The red box indicated the locus of deletion. (**E**) The expression of miR-487b-3p and *IRS1* mRNA in the miR-487b-3p KO clone. (**F**,**G**) Western blot analysis result of PI3K, phosphor-Akt (p-Akt), and t-Akt in miR-487b-3p KO myoblasts. (**H**,**I**) The protein expression of IRS1, PI3K, phosphor-Akt (p-Akt), and t-Akt in miR-487b-3p KO myoblasts transfected with siNC or siIRS1. Data are shown by mean ± SEM of three independent experiments. * *p* < 0.05; ** *p* < 0.01.

Previous studies have reported that *IRS1* enhances gene transcription by binding to RNA polymerases I and II, which is beneficial to cell proliferation and viability. We therefore hypothesized that miR-487b-3p may regulate goat myoblast growth through the IRS1/PI3K/Akt signal pathway. To verify this hypothesis, we first examined PI3K and Akt protein expression after *IRS1* knockdown using western blot. The results showed that siRNA-mediated *IRS1* knockdown led to significantly reduced PI3K and phosphor-Akt protein expression in goat myoblasts (*p* < 0.01, Figure 6A,B). We next sought to explore the molecular mechanism underlying how miR-487b-3p affects the IRS1/PI3K/Akt signaling pathway. We constructed an miR-487b-3p knockout primary goat myoblast clone with a deletion of seven nucleotides using CRISPR/Cas9 technology and our previously reported RPG surrogate reporter-based screening (Figure 6C, Figures S1 and S2). qRT-PCR analysis showed that miR-487b-3p expression was drastically reduced, while *IRS1* mRNA expression was significantly increased in the miR-487b-3p KO clone (*p* < 0.01, Figure 6E). In parallel, we found that the protein expression of IRS1, PI3K, and phosphor-Akt was increased in miR-487b-3p KO myoblasts (Figure 6F,G). To further investigate the function of the *IRS1* gene, the siRNA rescue experiment was conducted. The miR-487b-3p KO myoblasts were transfected with siIRS1 against the *IRS1* gene. The results demonstrated that *IRS1* silencing by siIRS1 in miR-487b-3p KO myoblasts also suppressed the PI3K/Akt signaling pathway (Figure 6H,I). In conclusion, these results revealed that miR-487b-3p is a negative regulator to inhibit goat myoblast growth, and affects the PI3K/Akt signaling pathway by modulating the *IRS1* gene.

## 3. Discussion

Skeletal muscle development is a well-coordinated biological process that is regulated by evolutionarily conserved networks of myogenic transcription factors [2,26]. Recently, an increasing number of reports have indicated that miRNAs are critical in the regulation of animal myogenesis [11,27,28]. A previous study using high-throughput sequencing declared that there was significantly higher miR-487b-3p expression in skeletal muscle tissues than other examined tissues [18]. In our experiments, we aimed to investigate the effect of miR-487b-3p on myogenesis in primary goat myoblasts. First, the expression profile of miR-487b-3p in different lamb or adult goat tissues was illustrated. We observed markedly higher miR-487b-3p expression in skeletal muscle than other tissues, which was consistent with previous studies [18,29]. Then, the association of miR-487b-3p with goat myoblast proliferation and differentiation was further verified. Additionally, miR-487b-3p has a highly conserved sequence among different species (*sheep*, *bovine*, *human*, *mouse*, and *rat*), indicating that miR-487b-3p may possess critical biological functions on the regulation of evolutionarily conservative genes. These results suggest that miR-487b-3p might be a muscle-related miRNA, similar to other previously reported muscle-specific miRNAs.

As a member of the miR-487b family, miR-487b-3p has previously been identified as a tumor-related miRNA that might play an independent role in glioneuroma [30]. Some studies have reported miR-487b-3p as a tumor suppressor that inhibits osteosarcoma chemoresistance and metastasis by targeting ALDH1A3 [31]. miR-487b may participate in the interaction between GRM3 and TGF-β, and restrain the proliferation and metastasis of CRC cells [32]. Moreover, miR-487b-3p was also reported to be capable of preventing cell proliferation and differentiation in C2C12 myoblasts by targeting *IRS1* [18]. In this study, the results of the CCK-8 and EdU assays both showed that miR-487b-3p upregulation and downregulation, respectively, repressed and promoted goat myoblast proliferation. Flow cytometry analysis revealed that miR-487b-3p mimic transfection caused proliferating myoblasts to be arrested at G1 phase, whereas miR-487b-3p inhibitor transfection caused a strong increase in S-phase myoblasts compared with the negative control. In addition, the mRNA and protein expression of proliferation marker genes (*PCNA*, *CDK2*, and *p27*) showed significant changes upon miR-487b-3p overexpression or knockdown. All these results suggest that miR-487b-3p can inhibit goat myoblast proliferation. Additionally, we found that miR-487b-3p also suppresses goat myoblast differentiation by analyzing critical regulators for myogenesis (*MyoG* and *MyHC*) and myotube formation. This is consistent with the previously reported results in the murine skeletal muscle cell line C2C12 [18]. Hence, miR-487b-3p may act as a key negative regulator of myogenic proliferation and differentiation in goats.

Identification of miRNA targets is essential for a comprehensive understanding of miRNA-mediated gene regulation. It has long been known that miRNAs bind to the 3′UTR of target mRNA genes and consequently lead to the degradation or translation inhibition of mRNAs [33,34]. Additionally, the molecular functions of miR-487b-3p have been demonstrated in a variety of cancer cells. In this study, we identified specific miR-487b-3p target genes using TargetScan and miRDB analysis software. We next demonstrate that *IRS1* is a direct target gene of miR-487b-3p by performing a series of experiments including dual luciferase assay, gain- or loss-of-function analysis, and western blot. Additionally, using siRNA to knock down the *IRS1* expression in goat myoblast, cell proliferation was significantly inhibited. These findings were in accordance with previously reported results. Altogether, these findings show that *IRS1* is one target gene of miR-487b-3p.

Previous studies indicated that the PI3K/Akt pathway is a pivotal signal transduction pathway involved in the regulation of cell proliferation, adhesion, and differentiation [35,36]. Furthermore, several studies have reported that dysregulated expression of miRNAs may be part of a feed-forward mechanism stimulating skeletal muscle growth and development such as miR-432 suppressing mouse myoblast proliferation and differentiation by inhibiting E2F3 and the PI3K/Akt pathway [37], and miR-21 targets TGFβ1 via the PI3K/Akt/mTOR signaling in development of the skeletal muscle of pig [38]. In this study, the targeting relationship between *IRS1* and miR-487b-3p was validated in goat myoblast. IRS1 is the most important representative of the IRS protein family and the critical factor in the PI3K/Akt signaling pathway. It has already been acknowledged that *IRS1* is a critical mediator of PI3K/Akt and AMPK signaling in the regulation of muscle growth and metabolism [39,40,41]. We therefore speculated that there may be important regulatory relationships between miR-487b-3p and *IRS1*, resulting in the alteration of myogenesis activity. Meanwhile, we found that the PI3K/Akt signaling pathway was suppressed by *IRS1* knockdown. Next, we succeeded with the knockout of miR-487b-3p in goat myoblast by introducing indels into its seed sequence using CRISPR/Cas9. Further studies demonstrated that siIRS1 could rescue the inhibition of activation of the PI3K/Akt signaling pathway. Further studies demonstrated that knockdown *IRS1* was able to rescue inhibition of PI3K/Akt signaling pathway activation by miR-487b-3p KO. These results revealed that miR-487b-3p may be a potent inhibitor of myogenesis in primary goat myoblasts by relieving its inhibition of IRS1/PI3K/Akt pathways.

In conclusion, we have demonstrated that miR-487b-3p inhibits goat myoblast proliferation and differentiation by inhibiting *IRS1* through the PI3K/Akt signaling pathway. To the best of our knowledge, this is the first research that uses the CRISPR/Cas9 system and surrogate reporter system to study the biological function of miRNA in primary goat myoblast. In addition, these results provide novel insights into the function of miR-487b-3p in goat myoblast and will contribute to our understanding of the growth mechanism of goat skeletal muscle.

## 4. Materials and Methods

### 4.1. Animal Tissue Sample Collection

All the procedures of animal experimentation in this study strictly followed the protocol approved by the Institutional Animal Care and Use Committee (IACUC) of Northwest Agricultural and Forestry University (NWAFU) (permit number: 15−516, date: 9-13-2015). The animals were then sacrificed by exsanguination following anesthesia. Heart, liver, spleen, lung, kidney, and muscle tissue (leg muscle tissue) samples were collected from three lamb goats (90 days old) and three adult goats (two years old). All samples were collected in sterile conditions. The tissues were then rinsed with PBS, flash-frozen in liquid nitrogen, and stored at −80 °C for further experiments.

### 4.2. Cells Culture

Primary goat myoblasts were isolated from semitendinosus tissues of the lamb goat, and were purified and cultured according to previously described protocol [42]. Briefly, cells were cultured in growth medium containing DMEM/F12 basic medium (Gibco, Grand Island, NY, USA), 20% fetal bovine serum (FBS, Gibco, Grand Island, NY, USA), and antibiotics (100 U/mL penicillin and streptomycin) (15140-122, Invitrogen Corp, Waltham, MA, USA) in 5% CO_2_ at 37 °C. For myoblast differentiation, we used differentiation media: the media were switched to a differentiation medium containing DMEM/F12 basic medium and 2% horse serum (Gibco, Grand Island, NY, USA). Additionally, HEK293T cells were grown in DMEM (Gibco, Grand Island, NY, USA) with 10% FBS. Culture medium was changed every day.

### 4.3. RNA Isolation and Quantitative Real-Time Polymerase Chain Reaction (qRT-PCR)

Total RNA was extracted from goat tissue samples or cultured cells using the reagent Trizol (Takara, Dalian, China). The concentration of total RNA measured by NanoDrop2000 (Thermo, San Jose, CA, USA) and the quality was checked by denaturing agarose gel electrophoresis. The cDNA synthesis was performed with reverse transcription kits (Takara, Dalian, China). For quantification of miR-487b-3p expression, miRNA specific complementary DNA was generated using miRNA stem-loop-specific primers (Table 1). The cDNA generated was stored at −20 °C for subsequent usage.

qRT-PCR was performed using TB green premix Ex taq II (Takara, Dalian, China) on a Light Cycler 96 real-time system (Roche, Basel, Switzerland) with a reaction volume of 25 μL. Each sample was carried out in triplicate and repeated for three times at least. The mRNA levels of all coding genes were normalized to the housekeeping gene glyceraldehyde-3-phosphate dehydrogenase (*GAPDH*), which was used as an internal standard. On the other hand, the expression level of miR-487b-3p was quantified with 18S RNA as the reference. The relative gene expression was analyzed using the comparative threshold cycle (CT) method (2^−∆∆Ct^). The primers of the target genes are listed in Table 1.

### 4.4. RNA Oligonucleotides

The miR-487b-3p mimics, mimics NC, miR-487b-3p inhibitors, inhibitors NC, and *IRS1* siRNA in this study were designed and synthesized by Sangon Biotech Co., Ltd., (Shanghai, China). The detailed sequences are shown in Table 2.

### 4.5. Cell Transfection

Primary goat myoblast cells were incubated in 6-well plates for 18–24 h until the cell density reached 70–80% confluence prior to transfection. miR-487b-3p overexpression and knockdown were achieved by transfecting myoblasts with miRNA mimics or inhibitors, respectively. In addition, RNA interference was performed by transfecting siRNA fragments of *IRS1* and a scrambled siRNA control. Transfection was performed using lip2000 reagent (Invitrogen, Carlsbad, CA, USA) according to the manufacturer’s protocols. All experiments were performed in three independent experiments with at least three replicates. Finally, the cells were harvested for RNA and protein extraction 48 h after transfection.

### 4.6. Cell Counting Kit-8 (CCK-8) Assays

The myoblast cells were seeded at a density of 1 × 10^3^ cells per well into a 96-well plate using a hemocytometer (Inno-Alliance Biotech, Wilmington, DE, USA). Three independent biological replicates for each treatment were conducted. The cells were switched to the medium with 10% CCK-8 reagent (DOJINDO, Kumamoto, Japan) at the time of harvest and incubated for another 4 h at 37 °C, which was followed by the absorbance measurement at 450 nm using a SYNERGY/H1 microplate reader (BioTek, Winooski, VT, USA). The intensity of the color was directly proportional to the number of viable cells in the sample.

### 4.7. 5-Ethynyl-2-deoxyuridine (EdU) Imaging Assays

The EdU imaging assay was carried out using the 488 EdU Click Proliferation Kit (Beyotime, Nantong, Jiangsu, China) according to the manufacturer’s instructions. Myoblasts were incubated with EdU (50 mM) in the growth culture medium for 2 h. Following incorporation of EdU and fixation, myoblast cells were subjected to Click-iT reaction for 30 min in the dark to add biotin to the EdU, and the cell nuclei were stained with 6-diamidino-2-phenylindole (DAPI) (Invitrogen) for 10 min. Afterward, the cells were visualized by using a cell imaging multifunctional detection system (BioTek, Winooski, VT, USA) and the data were analyzed with ImageJ software. The ratio of EdU-positive cells was calculated as (EdU-positive cells/DAPI-stained cells) × 100%.

### 4.8. Flow Cytometric Analysis

The myoblast cells were seeded in a 6-well plate at a density of 5 × 10^5^ cells per well. After 48 h of transfection, cells were washed three times with PBS and harvested. Then, the cells were collected and fixed in cold 70% ethanol at 4 °C overnight. Propidium iodide (PI) staining was performed using a PI Staining Kit (Beyotime, Nantong, Jiangsu, China) according to the manufacturer’s instructions. Finally, the cells were re-suspended in 1 mL PBS and analyzed by a BD FACSAria™ III flow cytometry system (Franklin Lakes, NJ, USA).

### 4.9. Bioinformatics Analysis for Target Genes Prediction

The sequences of miRNAs were obtained from the miRNA Registry miRBase (http://www.mirbase.org/, accessed on 1 September 2021) and the 3’UTR sequence of *IRS1* were downloaded from NCBI (https://www.ncbi.nlm.nih.gov/, accessed on 1 September 2021). The target genes of miRNA were predicted by TargetScan (http://www.targetscan.org, accessed on 1 September 2021) and miRDB.

### 4.10. Dual-Luciferase Reporter Assay

The 3΄UTR of goat *IRS1* mRNA were amplified from myoblast cDNA with the primers shown in Table 3. The wild type or mutant 3′UTR sequences of *IRS1* were cloned into the psi-CHECK2 vector (Promega, Mannheim, Germany) with restriction sites of Xho I and Not I, respectively. HEK293T cells were seeded into 48-well plates at a density of 1 × 10^4^ cells per well. The cells were co-transfected with 250 ng of the wild type (WT, psi-CHECK2-IRS1-3′UTR) or the mutant (Mutant, psi-CHECK2-IRS1-3’UTR-mut) 3’UTR plasmids and 100 nM miR-487b-3p mimics or negative control (NC) using the Lipofectamine 2000 reagent following the manufacturer’s protocol. The primer sequences are shown in Table 2. After transfection for 48 h, cells were harvested and lysed in passive lysis buffer (Promega, Madison, WI, USA), and the dual-luciferase activity assay was performed according to the manufacturer’s instructions. Firefly luciferase and Renilla luciferase assays was performed using a SYNERGY/H1 microplate reader (BioTek, Winooski, VT, USA), and the value of firefly luciferase activity/Renilla luciferase activity was analyzed.

### 4.11. Protein Extraction and Western Blot Analysis

The total protein was extracted using RIPA lysis buffer (Solarbio, Beijing, China) with a protease inhibitor mix (Roche, Mannheim, Germany) after washing the cells with PBS three times. The protein concentration was determined using the BCA Protein Assay Kit (Thermo, San Jose, CA, USA). Thirty μg of protein for each sample was separated by SDS-PAGE (sodium dodecyl sulfate polyacrylamide gel electrophoresis) followed by transferring onto a PVDF (polyvinylidene fluoride) membrane (Millipore, Burlington, MA, USA). The membrane was blocked with 5% defatted milk (BD, Franklin Lakes, NJ, USA) in TBST (Tris-buffered saline with Tween 20) buffer for 1 h at room temperature, and then incubated with antibodies (1:1000) against anti-PCNA, anti-PI3K (Cell Signaling Technology, Danvers, MA, USA), anti-CDK2 (SAB), anti-MyoG, anti-p27 (Santa Cruz, Santa Cruz, CA, USA), anti-Akt, anti-p-Akt (Cell Signaling Technology), and anti-GAPDH (diluted 1:2000) (Bioss, Beijing, China) at 4 °C overnight. The following day, each membrane was washed three times with TBST for 10 min each time. Subsequently, the membrane was incubated with HRP-conjugated secondary antibodies (anti-mouse IgG or anti-rabbits IgG) (Bioss, Beijing, China) diluted at 1:2000. Chromogenic reaction was performed using an enhanced chemiluminescent (ECL) western blot substrate (Advansta, Menlo Park, CA, USA) and was detected by a Sage Capture TM System (BioTek, Winooski, VT, USA). ImageJ software was used for densitometric analysis.

### 4.12. Immunofluorescence

After four days of differentiation, myoblast cells were fixed and immunofluorescent staining performed. Cells were fixed with 4% paraformaldehyde (PFA) for 30 min and permeabilized with 0.5% Triton X-100 (Sigma, St. Louis, MO, USA) for 15 min at room temperature. After washing with PBS, the cells were blocked with PBS containing 5% bovine serum albumin (BSA) for 1 h. Then, the myoblast cells were incubated with specific antibodies against MyHC (diluted 1:200) (Santa Cruz) overnight at 4 °C, and washed with PBS (3 × 5 min) at room temperature. Subsequently, the cells were incubated with FITC-conjugated anti-mouse IgG (1:50) (Bioss, Beijing, China) for 1 h at room temperature. Following three washes with PBS, the cell nuclei were co-stained with 4, DAPI in the dark for 2 min. Three biological repeats were performed for each treatment. The field of vision was randomly selected for analysis in each biological repeat. Next, the number of DAPI-stained nuclei was calculated by ImageJ software and the nuclei in myotubes was calculated manually. The differentiation index = The number of nuclei in myotubes (MyHC-positive cells)/Total number of nuclei under visual field.

### 4.13. Statistical Analysis

All statistics were analyzed by GraphPad Prism 6.0 software and the data were expressed as the “means ± SEM”. The statistical significance of difference was assessed by the unpaired Student’s t-test for two group comparisons or a one-way ANOVA for more than two groups. The difference was considered significant when the corresponding *p* value was less than 0.05 (*) or 0.01 (**).

## Figures and Tables

**Table 1:** Primer information for miRNA and mRNA quantitative reverse transcription.

Gene	Primer Name	Primer Sequence (5′ to 3′)	Reference
*miR-487b-3p*	Stem-loop	GTCGTATCCAGTGCAGGGTCCGAGGTATTCGCACTGGATACGACAAGTGG	[18]
	miR-487b-3p-F	CGGGCAATCGTACAGGGT	This manuscript
	miR-487b-3p-R	CAGTGCAGGGTCCGAGGTAT	
*18S-rRNA*	18S-rRNA-F	GTGGTGTTGAGGAAAGCAGACA	[18]
	18S-rRNA-R	TGATCACACGTTCCACCTCATC	
*IRS1*	IRS1-F	GTAGTGGCAAACTCCTGTCTTGT	This manuscript
	IRS1-R	GAGTAGTAGGAGAGGACGGGCT	
*PCNA*	PCNA-F	CGCTTAAGGATCTCATCAATGAG	This manuscript
	PCNA-R	GTTACGGTCGCAGCGGTAAG	
*CDK2*	CDK2-F	TCATGGATGCCTCTGCACTC	[42]
	CDK2-R	CTCTGGCTAGTCCGAAGTCTG	
*p27*	p27-F	CGGCGGTGCCTTTACTT	[42]
	p27-R	GCAGGTCGCTTCCTTATCC	
*MyoG*	MyoG-F	GGACCCTACAGATGCCCACAA	This manuscript
	MyoG-R	TTGGTATGGTTTCATCTGGG	
*MyHC*	MyHC-F	GTGAAGGAGGACCAGGTGTTG G	[42]
	MyHC-R	GTTGATGGTGACGCAGAAGAG	
*GAPDH*	GAPDH-F	CCACGCCATCACTGCCACCC	[42]
	GAPDH-R	CAGCCTTGGCAGCGCCAGTA	

**Table 2:** RNA oligonucleotides in this article.

Name	Sense Sequence (5′-3′)
Mimics	AAUCGUACAGGGUCAUCCACUU
Mimics NC	UUCUCCGAACGUGUCACGUTT
Inhibitors	AAGUGGAUGACCCUGUACGAUU
Inhibitors NC	CAGUACUUUUGUGUAGUACAA
siIRS1	GCAUGCACAAGCGAUUCUUTT
	AAGAAUCGCUUGUGCAUGCTT
siNC	UUCUCCGAACGUGUCACGUTT
	ACGUGACACGUUCGGAGAATT

**Table 3:** The primers used to construct the plasmid.

Name	Primer Name	Primer Sequence (5′ to 3′)
Wild-IRS1-3′UTR	Wild-IRS1-F	cgg***CTCGAG***CAGCAAATCCTCCTTTAACTC
	Wild-IRS1-R	aat***GCGGCCGC***GCACGATATACAACGTGCAG
Mutant-IRS1-3′UTR	Mutant-IRS1-F	CTCAGTAGATGGGCTAATGCACCC
	Mutant-IRS1-R	GAAATGGGTGCCGATTACCATCTAC

Note: The protective bases are indicated in lowercase letters; The *Xho* I and *Not* I restriction sites are indicated in uppercase italic characters.

## Supplementary Materials

The following are available online at https://www.mdpi.com/article/10.3390/ijms23010115/s1.
