# Supplementary material for: chi-miR-487b-3p Inhibits Goat Myoblast Proliferation and Differentiation by Targeting IRS1 through the IRS1/PI3K/Akt Signaling Pathway
Source: Int J Mol Sci. 2021 Dec 23;23(1):115. doi: 10.3390/ijms23010115 (PMC8745444; doi:10.3390/ijms23010115)

Supplementary Materials:

Suppl. Figure 1. Primary goat myoblast treated with 0 μg/mL, 0.5 μg/mL, 0.75 μg/mL and 1.0 μg/mL puromycin for 72 hours and the cell death condition was examined by microscope. The optimal puromycin concentrations was 0.75 μg/mL.

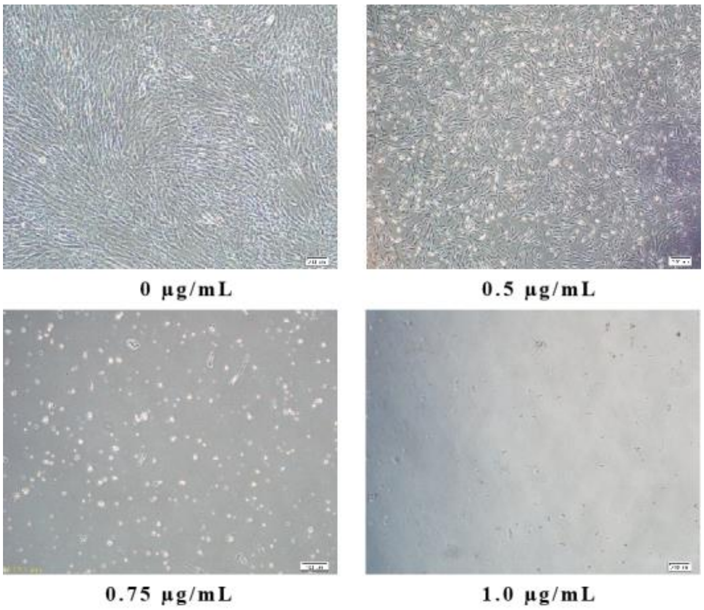

Suppl. Figure 2.

After co-transfected with the CRISPR/Cas9 and RPG surrogate reporter vectors, the primary goat myoblast treated with 0.75 μg/mL puromycin for 0 hours or 72 hours and the cell condition was examined by microscope.

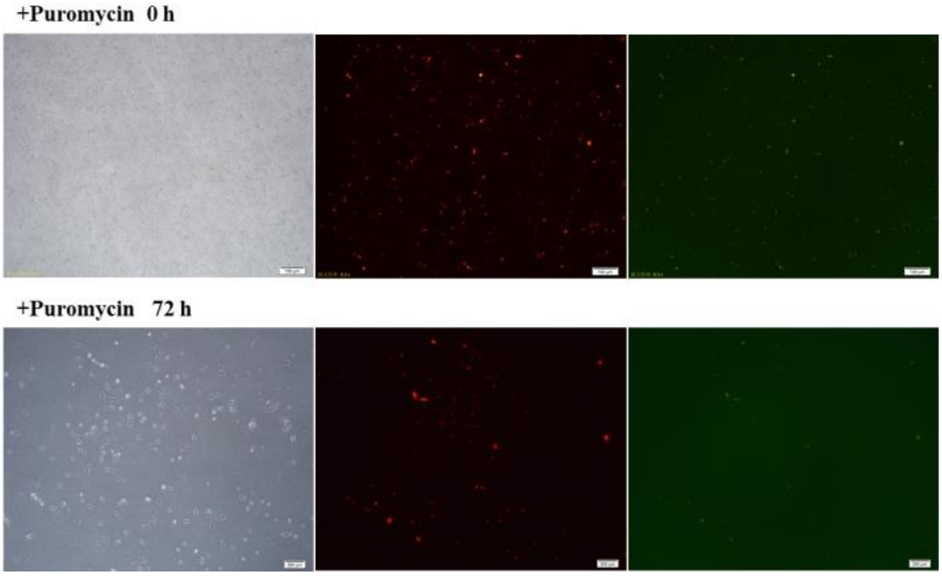

Supplement: Supplementary file 1 [file ijms-23-00115-s001.zip › ijms-1473391-supplementary.pdf]
